# P082 When polymyalgia rheumatica wasn’t the whole story: PET-confirmed occult large vessel giant cell arteritis preceding primary middle ear diffuse large B-cell lymphoma

**DOI:** 10.1093/rap/rkag113.112

**Published:** 2026-11-03

**Authors:** Julia McEvoy, Mark Lloyd, James Mitchell, Asha Aggarwal

**Affiliations:** Frimley Park Hospital, Frimley, United Kingdom; Frimley Park Hospital, Frimley, United Kingdom; Frimley Park Hospital, Frimley, United Kingdom; Frimley Park Hospital, Frimley, United Kingdom

## Abstract

**Introduction:**

We describe a patient with polymyalgia rheumatica (PMR) and PET-confirmed silent aortitis who subsequently developed a primary middle ear diffuse large B-cell lymphoma (DLBCL) 17 months after initial presentation. This case highlights the question of who to screen for large vessel involvement in PMR and giant cell arteritis (GCA), and the need to keep an open mind when new symptoms arise during follow up. We discuss the relationship between lymphoma and rheumatic disease.

**Case description:**

A 79-year-old woman presented in February 2024 with a three-month history of progressive fatigue, profound morning stiffness involving the shoulder and pelvic girdles, finger pain, and weight loss. She had no headache, scalp tenderness, jaw or tongue claudication or visual disturbance. She had a recent history of gastric ulcer (Forest 3). She had painful restricted shoulder movement, with wrist and PIP swelling. Temporal arteries were difficult to feel but not tender.

CRP was 102mg/L with an iron deficiency anaemia. Other routine bloods were normal, RF, CCP, ANA negative, urate 229umol/l. A clinical diagnosis of PMR was made. PET-CT scan was arranged with a view to avoiding steroids if large vessel vasculitis (LVV) was excluded. However, it showed increased metabolic activity in the thoracic and abdominal aorta and proximal large vessel branches particularly the subclavian vessels, consistent with large-vessel GCA. Bilateral trochanteric and ischial bursitis was also noted, and bilateral axillary lymphadenopathy. Prednisolone 40mg daily with omeprazole and IV zoledronate were started. Methotrexate was started but discontinued following an allergic reaction. Between October 2024 and July 2025 CRP fluctuated between 5-32 mg/L despite prednisolone dose adjustments. Tocilizumab was started, achieving good control of symptoms and CRP normalised.

In July 2025 she developed progressive left-sided hearing loss and left VII palsy. Imaging and biopsies demonstrated diffuse large B-cell lymphoma arising within the left middle ear and transverse/sigmoid sinus thrombosis. Staging investigations demonstrated localised extranodal disease without axillary node enlargement. R-mini-CHOP was commenced followed by radiotherapy.

By April 2026 she was on prednisolone 2mg with no PMR symptoms but CRP was still raised at 24. Repeat PET-CT showed persistent thoracic aortic vasculitis and incidentally identified a 18mm left lung lesion with biopsy result pending. Prednisolone was increased to 20mg daily. The patient is concerned tocilizumab may predispose to lymphoma development.

**Discussion:**

PMR and GCA represent a spectrum of inflammatory disease sharing common pathogenic mechanisms involving innate and adaptive immune activation through interleukin-6 signalling. Approximately 20% of patients with PMR develop GCA. Large-vessel involvement may occur without classical cranial manifestations.

2026 European guidelines recommend referring all patients with PMR to Rheumatology specialists. The BSR GCA guidelines highlight the role of PET-CT for diagnosing LVV and identifying neoplastic or infective conditions that may mimic or coexist with PMR.

Another diagnostic question is whether the initial PMR syndrome represented genuine inflammatory vasculitis or a paraneoplastic process. Several reports describe lymphoma presenting with PMR-like symptoms, highlighting the need to reconsider alternative diagnoses when inflammatory markers remain elevated, clinical features are atypical, or treatment response is suboptimal. PET-CT performed early in this patient’s disease course demonstrated active large-vessel aortitis and bursitis consistent with PMR with GCA without evidence of malignancy. This supports PMR with GCA rather than lymphoma masquerading as PMR.

The relationship between PMR/GCA and malignancy remains controversial. Although chronic inflammatory diseases such as rheumatoid arthritis are associated with an increased lymphoma risk, population-based studies have not demonstrated a convincing increase in PMR/GCA. Previously reported associations may reflect diagnostic overlap, surveillance bias or coincidental occurrence in an elderly population.

Primary middle ear diffuse large B-cell lymphoma is exceptionally rare and often mimics chronic otitis media, delaying diagnosis until biopsy. Our patient required multiple biopsies to reach diagnosis, highlighting the need to persevere when initial investigations are inconclusive. There is no current evidence demonstrating causation between IL-6 inhibition and lymphoma. The temporal sequence and early PET findings favour two distinct disease processes, although shared age-related immune dysregulation cannot be excluded. This case underscores the importance of diagnostic vigilance for new pathology during follow-up, even when inflammatory disease appears well controlled.

**Key learning points:**

1. Polymyalgia rheumatica and giant cell arteritis exist along a disease spectrum. The challenge is identifying patients with PMR and silent large vessel involvement who may be undertreated as PMR alone.

2. It is important in patients with PMR-like symptoms to distinguish rheumatic disease from an underlying paraneoplastic syndrome. In this case, early PET-confirmed large-vessel vasculitis without evidence of malignancy supports PMR preceding lymphoma rather than occult lymphoma masquerading as PMR.

3. New or evolving symptoms during immunomodulatory therapy should not automatically be attributed to active rheumatic disease or treatment-related adverse effects. Infection, malignancy and alternative diagnoses should be actively reconsidered throughout follow-up.

4. Current epidemiological evidence does not demonstrate a convincing increase in lymphoma risk among patients with PMR or GCA, neither does available data support a causal association between tocilizumab therapy and lymphoma development.

